# Mortality patterns of patients with tonsillar squamous cell carcinoma: a population-based study

**DOI:** 10.3389/fendo.2023.1158593

**Published:** 2023-12-07

**Authors:** Jia Wang, Xiaolin Li, Dongdong Niu, Jiasheng Huang, Enlin Ye, Yumei Zhao, Suru Yue, Xuefei Hou, Jiayuan Wu

**Affiliations:** ^1^ Clinical Research Service Center, Affiliated Hospital of Guangdong Medical University, Zhanjiang, Guangdong, China; ^2^ Guangdong Engineering Research Center of Collaborative Innovation of Clinical Medical Big Data Cloud Service in Western Guangdong Medical Union, Affiliated Hospital of Guangdong Medical University, Zhanjiang, Guangdong, China

**Keywords:** tonsillar squamous cell carcinoma, second primary malignancies, nomogram, competitive risk model, risk factor

## Abstract

**Objective:**

Tonsillar squamous cell carcinoma (TSCC) and second primary malignancies (SPMs) are the most common causes of mortality in patients with primary TSCC. However, the competing data on TSCC-specific death (TSD) or SPM-related death in patients with TSCC have not been evaluated. This study aimed to analyze the mortality patterns and formulate prediction models of mortality risk caused by TSCC and SPMs.

**Methods:**

Data on patients with a first diagnosis of TSCC were extracted as the training cohort from the 18 registries comprising the Surveillance, Epidemiology, and End Results (SEER) database. A competing risk approach of cumulation incidence function was used to estimate cumulative incidence curves. Fine and gray proportional sub-distributed hazard model analyses were performed to investigate the risk factors of TSD and SPMs. A nomogram was developed to predict the 5- and 10-year risk probabilities of death caused by TSCC and SPMs. Moreover, data from the 22 registries of the SEER database were also extracted to validate the nomograms.

**Results:**

In the training cohort, we identified 14,530 patients with primary TSCC, with TSCC (46.84%) as the leading cause of death, followed by SPMs (26.86%) among all causes of death. In the proportion of SPMs, the lungs and bronchus (22.64%) were the most common sites for SPM-related deaths, followed by the larynx (9.99%), esophagus (8.46%), and Non-Melanoma skin (6.82%). Multivariate competing risk model showed that age, ethnicity, marital status, primary site, summary stage, radiotherapy, and surgery were independently associated with mortality caused by TSCC and SPMs. Such risk factors were selected to formulate prognostic nomograms. The nomograms showed preferable discrimination and calibration in both the training and validation cohorts.

**Conclusion:**

Patients with primary TSCC have a high mortality risk of SPMs, and the competing risk nomogram has an ideal performance for predicting TSD and SPMs-related mortality. Routine follow-up care for TSCC survivors should be expanded to monitor SPMs.

## 1 Introduction

Oropharyngeal carcinomas are mostly composed of two subsites, namely, the palatine tonsils and the base of the tongue (1). Approximately 90% of oropharyngeal tumors are squamous cell carcinomas (SCC) (2). According to an epidemiological study, tonsillar squamous cell carcinoma (TSCC) represents approximately 15%–20% of all oropharyngeal SCCs in the United States (3). The incidence of tonsil cancer in England continued to increase between 1985–2006 (4). The burden of TSCC has also increased in most regions of the United States between 2000 and 2014 regardless of regional socioeconomic status (5). Moreover, the incidence of TSCC has increased in young adults possibly because of HPV16 viral infection (6, 7).

The typical symptoms of TSCC include swallowing difficulties, unilateral pain in the throat and ear, and lumps in the neck (8). Most patients with TSCC are treated with multimodal treatment combined with surgery, radiotherapy, and chemotherapy (9), and this process can increase the survival rate by 20% compared with those who received a single treatment modality (1). Traditional open surgical treatment, including mandibular incision and pharyngectomy, is likely to cause serious complications, such as speech, swallowing, and breathing disorders (10). Recently, as a relatively safe, effective, and minimally invasive treatment, transoral robotic surgery has been increasingly used in the treatment of TSCC (11), and the results are comparable to those of open surgery (12).

The survival rates of patients with TSCC in the United States have improved significantly over the past decades, with a 28%–60% improvement in five-year overall survival between 1980 and 2000 (13). Psychogios et al. observed a five-year overall survival (OS) rate of 66.0% for TSCC in 2000–2012. For most patients with TSCC, the improvement in survival means an increased risk of other diseases, including second primary malignancies (SPMs) and cardiovascular diseases (11). SPMs are the second leading cause of death in patients with oropharyngeal cancer, contributing to 69% of deaths in TSCC patients after 3 years of TSCC diagnosis (12). Although the pathogenesis of SPMs in TSCC is unclear, the patients experience a shortened survival period upon disease occurrence, and adolescents and young adults with SPMs have a worse survival outcome than those with only primary cancer (14). The understanding of mortality pattern after TSCC is critical for the improved management of patients with TSCC and reduced mortality. To the best of our knowledge, limited studies have focused on the mortality pattern of TSCC patients.

Nomogram has been widely used for the prognosis prediction of various cancers because of its simplicity, intuitiveness, and practicality (15), which can provide quantifiable measurements for an individual patient. The American Joint Committee on Cancer (AJCC) tumor staging system is the most common tool for predicting prognosis in patients with TSCC. However, such system depends on the anatomical extent of the cancer and do not consider clinicopathological characteristics and patient demographics, resulting in the unreliable assessment of individual patients. The validity of the nomogram has been proven and has been widely used in the prediction of various cancers, including gastric cancer (16) and breast cancer (17). However, most of these nomogram risk maps are developed based on Cox proportional risk models and do not consider competitive risk in cancer outcomes. The Cox model also ignores the mutually exclusive relationship between target and competing events, leading to the overestimation of true observations. The competing risk model is suitable for the processing of event data and can obtain approximately unbiased results that are close to the truth (18). The choice of survival analysis method is critical in the nomogram construction process. In the present study, we aimed to develop and validate nomograms to predict TSCC-specific death (TSD) and SPM-related mortality according to the Surveillance, Epidemiology and End Results (SEER) database by using a competing risk model.

## 2 Materials and methods

### 2.1 Data source

SEER*Stat software (version 8.4.1), which was obtained from the National Cancer Institute (available at https://seer.cancer.gov/seerstat/), was used to extract data from the National Cancer Institute (https://seer.cancer.gov/). The SEER database represents 28% of the US population and is the largest cancer database in the United States. Ethical approval was waived, and informed consent was not necessary, because SEER study data were anonymized and public.

In this study, we utilized data from 18 registries of the SEER database as the training cohort and data from 22 registries as the validation cohort. The training cohort was used to establish nomograms for predicting TSD and SPM-related mortality, while the validation cohort was used to verify the prediction models.

### 2.2 Study population

Primary tumor patients first diagnosed with TSCC were extracted from the SEER database (18 registries and 22 registries). The following International Classification of Diseases for Oncology, third edition (ICD-O-3) histological codes were used: 8070/3, 8071/3, 8072/3, and 8073/3. Primary site codes were used for the tonsillar fossa (C09.0), tonsillar pillar (C09.1), overlapping lesion of tonsil (C09.8), and tonsil (C09.9). Patients with incomplete data, age of less than 18 years, and survival time of less than 1 month were excluded. We extracted various determinants, such as year of diagnosis, gender, age at diagnosis, ethnicity, site of primary tumor, surgery, chemotherapy, radiotherapy, summary stage, histological grade, marital status, laterality, cause of death, and survival time, from the SEER database. The primary outcomes of interest were TSD and death from SPMs after diagnosis of TSCC. In the present study, tumor site was defined based on the SEER’s definition standard for SPMs, that is, the ICD-O-3 tissue coding of SPMs is different from that of TSCC. SPMs were defined as SPMs with an incubation period of more than 6 months after the diagnosis of primary TSCC (19).

### 2.3 Statistical analysis

This study had two notable events, namely, TSD and SPM-related death. TSD was defined as the period from the diagnosis to the death caused by TSCC or censoring, of which the competing event was defined as other causes of death rather than TSCC. SPM-related death was defined as the period from the diagnosis to the death caused by SPMs or censoring, of which the competing event was defined as other causes of death rather than SPMs.

In the training cohort, the crude cumulative incidence function (CIF) curves were plotted, and Gray’s test was carried out to identify differences in TSD and SPM-related mortality between subgroups (20). Univariate and multivariate fine and gray proportional sub-distributed hazard model analysis was then performed to identify the independent risk factors for TSD and SPMs-related mortality (21). Kaplan-Meier methods were also conducted to calculate the cumulative mortality rates of different causes. The risk factors with statistical significance (*P* value < 0.05) in multivariate analysis were selected to establish nomograms for predicting TSD and SPM-related mortality.

Nomogram performance in relation to the concordance index (*C*-index) and calibration curve in the training and then the validation cohort was quantified. The *C*-index was used to describe the difference between the true and predicted value of the model with values ranging from 0.5 (no discrimination) to 1.0 (perfect discrimination). In the calibration curve, the vertical axis represents the actual probability, while the horizontal axis represents the predicted probability. The actual probability/predicted value pairs follow a 45° line through the origin, indicating that a single nomogram is well calibrated (22). The R software version 4.1.2 (https://www.r-project.org) and the cmprsk, dplyr, QHScrnomo, and survival packages were used for all statistical analyses. A two-sided *P* value < 0.05 was considered statistically significant.

## 3 Results

### 3.1 Population characteristics

**Figure 1:**
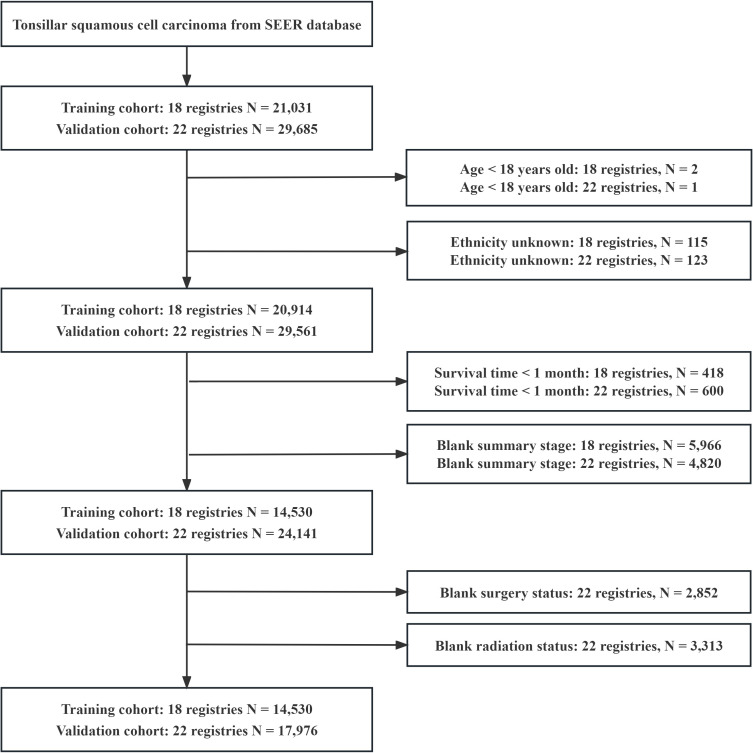
Flowchart of inclusion and exclusion criteria for enrolling patients.

**Figure 2:**
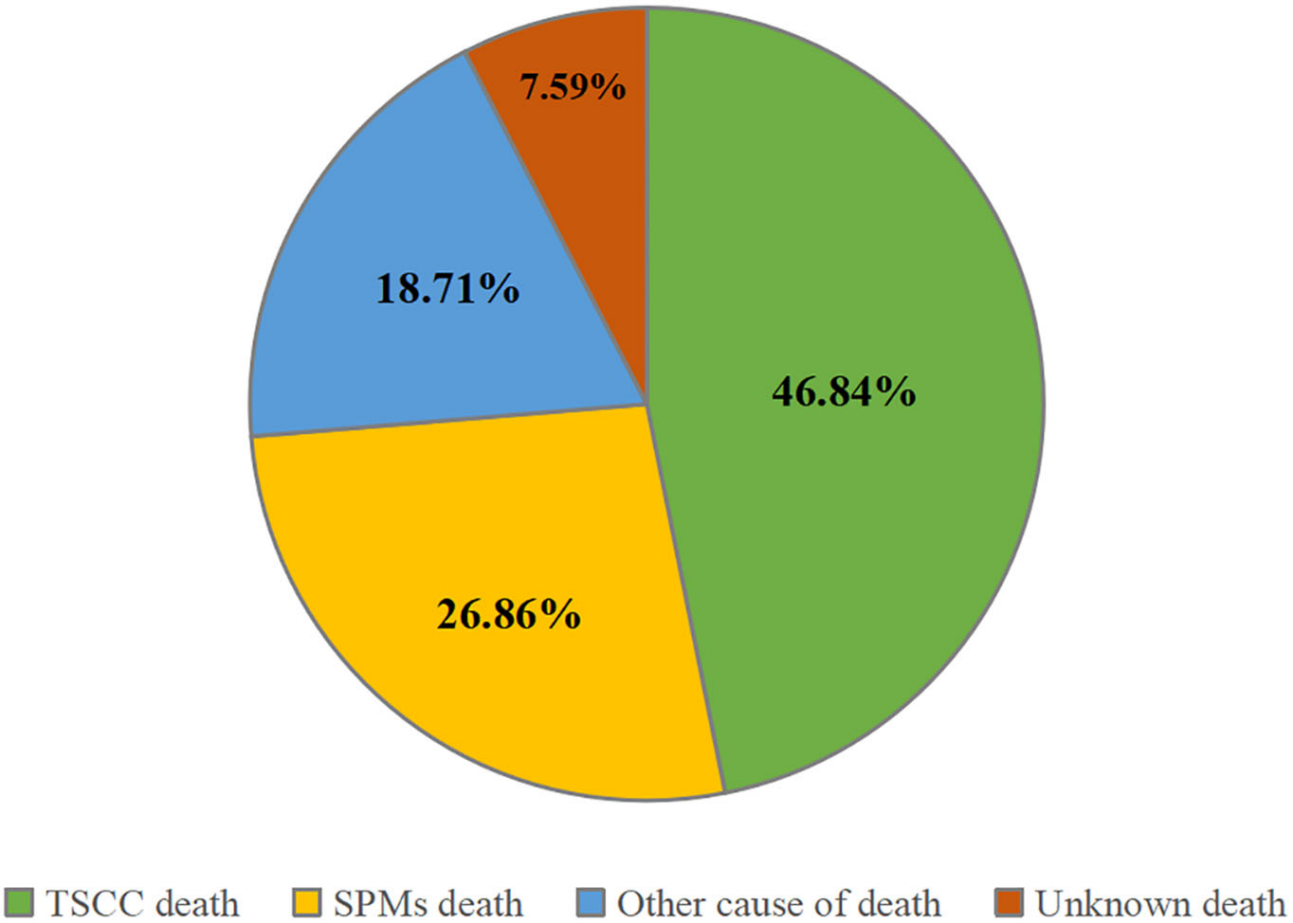
Distribution of mortality causes in tonsillar squamous cell carcinoma.

A total of 32,506 patients were eligible for the study, including 14,530 in the training cohort and 17,976 in the validation cohort (**Figure 1**). In the training cohort, most patients were white people (88.1%), male (82.0%), and treated with radiotherapy (84.6%). In the summary stage, the regional stage (70.3%) was predominant, followed by the distant stage (15.2%) and the localized stage (12.7%). The baseline characteristics of the training and validation cohorts are detailed in **Table 1**. During the follow-up period, TSCC accounted for 46.84% of all deaths, SPMs for 26.86%, other causes of death for18.71%, and unknown status for 7.59% in the training cohort (**Figure 2**). According to Kaplan-Meier survival analysis, the most common cause of SPM-related death was lung and bronchus, followed by larynx, esophagus, and non-melanoma skin (**Table 2**).

**Table 1:** Baseline characteristics of patients with tonsillar squamous cell carcinoma [n (%)].

Characteristics	Training cohort (n=14,530)	Validation cohort (n=17,976)
Ethnicity		
White	12794 (88.1)	15864 (88.2)
Black	1217 (8.4)	1523 (8.5)
Others	519 (3.5)	589 (3.3)
Gender		
Male	11911 (82.0)	14736 (82.0)
Female	2619 (18.0)	3240 (18.0)
Grade		
Well differentiated	650 (4.4)	825 (4.5)
Moderately differentiated	6054 (41.7)	7672 (42.7)
Poorly differentiated	7608 (52.4)	9270 (51.6)
Undifferentiated	218 (1.5)	209 (1.2)
Radiation		
Yes	12291 (84.6)	15171 (84.4)
None	2239 (15.4)	2805 (15.6)
Chemotherapy		
Yes	9672 (66.6)	12043 (67.0)
None	4858 (33.4)	5933 (33.0)
Surgery		
Yes	7641 (52.6)	9174 (51.0)
None/Unknown	6889 (47.4)	8802 (49.0)
Marital status		
Married	8251 (56.8)	10174 (56.6)
Single/Divorced	5512 (37.9)	6813 (37.9)
Unknown	767 (5.3)	989 (5.5)
Age (year)		
18-44	899 (6.2)	991 (5.5)
45-54	4326 (29.8)	5002 (27.8)
55-64	5558 (38.2)	6978 (38.9)
> 64	3747 (25.8)	5005 (27.8)
Laterality		
Left	7099 (48.9)	8735 (48.6)
Right	7305 (50.3)	9092 (50.6)
Bilateral	79 (0.5)	81 (0.5)
Others	47 (0.3)	68 (0.4)
Summary stage		
Localized	1849 (12.7)	2307 (12.8)
Regional	10212 (70.3)	12767 (71.0)
Distant	2206 (15.2)	2550 (14.2)
Unknown	263 (1.8)	352 (2.0)
Calendar year of diagnosis		
2000-2004	984 (6.8)	0
2005-2009	5331 (36.7)	5275 (29.3)
2010-2014	6806 (46.8)	7947 (44.2)
2015-2017	1409 (9.7)	4754 (26.5)
Primary site		
Tonsillar fossa	1970 (13.6)	2185 (12.2)
Tonsillar pillar	944 (6.5)	1100 (6.1)
Overlapping lesion of tonsil	149 (1.0)	218 (1.2)
Tonsil	11467 (78.9)	14473 (80.5)

**Table 2:** Cumulative mortality rates of different causes based on Kaplan-Meier analysis.

Time	All cause		TSCC		SPMs		Other cause		Unknown	
	Event	Mortality rate (%)	Event	Mortality rate (%)	Event	Mortality rate (%)	Event	Mortality rate (%)	Event	Mortality rate (%)
5-year	4495	0.313	2469	0.180	1180	0.097	586	0.051	260	0.022
10-year	5729	0.445	2780	0.221	1551	0.154	990	0.116	408	0.047

Time	Lung and Bronchus		Larynx		Esophagus		Non-Melanoma Skin		Other SPMs	
	Event	Mortality rate (%)	Event	Mortality rate (%)	Event	Mortality rate (%)	Event	Mortality rate (%)	Event	Mortality rate (%)
5-year	252	0.022	137	0.011	111	0.009	101	0.008	676	0.056
10-year	371	0.042	164	0.016	148	0.016	112	0.010	847	0.083

TSCC, tonsillar squamous cell carcinoma; SPMs, second primary malignancies.

### 3.2 Competing risk model

The CIF curves for TSD and SPM-related mortality are shown in **Supplementary Figures 1**, **2**, respectively. An increased risk of TSD and SPM-related death was observed in black people, male, patients without radiotherapy, patients who have not undergone surgery or whose surgical conditions are unknown, single or divorced, and distant stage. The results of subgroup analyses by gender and ethnicity were shown in **Supplementary Table 1**, male patients and blacks had higher mortality rates than other subgroups.

To explore the risk factors associated with TSD and SPM-related mortality, we used the fine and gray proportional sub-distributed hazard model to conduct univariate and multivariate analysis and the results are shown in **Tables 3**, **4**, respectively. Univariate analysis showed that ethnicity (*P* < 0.001), gender (*P* = 0.045), radiotherapy (*P* < 0.001), chemotherapy (*P* = 0.035), surgery (*P* < 0.001), marital status (*P* < 0.001), age (*P* < 0.001), summary stage (*P* < 0.001), and primary site (*P* < 0.001) were significantly associated with TSD. Moreover, ethnicity (*P* < 0.001), radiotherapy (*P* < 0.001), surgery (*P* < 0.001), marital status (*P* < 0.001), age (*P* < 0.001), summary stage (*P* < 0.001), and primary site (*P* = 0.002) were significantly associated with SPM-related mortality. Multivariate analysis revealed that ethnicity (*P* < 0.001), radiotherapy (*P* < 0.001), surgery (*P* < 0.001), marital status (*P* < 0.001), age (*P* < 0.001), summary stage (*P* < 0.001), and primary site (*P* = 0.002) are independent risk factors for TSD. Furthermore, ethnicity (*P* < 0.001), radiotherapy (*P* < 0.001), surgery (*P* < 0.001), marital status (*P* < 0.001), age (*P* < 0.001), summary stage (*P* < 0.001), and primary site (*P* < 0.001) are independent risk factors for SPM-related death.

**Table 3:** Univariate and multivariate competing risk analyses for cause-specific death in patients with tonsillar squamous cell carcinoma.

Characteristics	Univariate analysis			Multivariate analysis		
	SHR	*95% CI*	*P*-value	SHR	*95% CI*	*P*-value
Ethnicity						
White people	Reference			Reference		
Black people	1.755	1.520-2.030	< 0.001	1.333	1.198-1.483	< 0.001
Others	0.939	0.715-1.230	0.650	0.928	0.772-1.115	0.430
Gender						
Male	Reference					
Female	1.130	1.000-1.280	0.045			
Radiation						
Yes	Reference			Reference		
None	1.900	1.690-2.120	< 0.001	1.827	1.591-2.098	< 0.001
Chemotherapy						
Yes	Reference					
None	1.110	1.040-1.230	0.035			
Surgery						
Yes	Reference			Reference		
None/Unknown	2.450	2.220-2.700	< 0.001	2.057	1.833-2.307	< 0.001
Marital status						
Married	Reference			Reference		
Single/Divorced	1.660	1.510-1.830	< 0.001	1.521	1.421-1.629	< 0.001
Unknown	1.350	1.090-1.670	0.005	1.105	0.950-1.285	0.20
Age (years)						
18-44	Reference			Reference		
45-54	1.210	0.938-1.560	0.140	1.071	0.831-1.388	0.580
55-64	1.470	1.147-1.880	0.002	1.247	0.971-1.603	0.084
> 64	2.620	2.046-3.360	< 0.001	1.924	1.495-2.476	< 0.001
Summary stage						
Localized	Reference			Reference		
Regional	1.395	1.236-1.469	< 0.001	1.240	1.115-1.379	< 0.001
Distant	2.392	2.261-3.360	< 0.001	1.917	1.687-2.179	< 0.001
Unknown	1.601	1.130-2.023	< 0.001	1.414	1.134-1.765	< 0.001
Primary site						
Tonsillar fossa	Reference			Reference		
Tonsillar pillar	0.960	0.788-1.170	0.690	1.041	0.849-1.276	0.700
Overlapping lesion of tonsil	1.023	0.669-1.563	0.920	0.973	0.626-1.513	0.900
Tonsil	0.679	0.599-0.769	< 0.001	0.814	0.712-0.931	0.002
Laterality						
Left	Reference					
Right	0.933	0.849-0.930	0.015			
Bilateral	1.181	0.662-2.110	0.570			
Others	1.408	0.718-2.760	0.320			

**Table 4:** Univariate and multivariate competing risk analyses of death due to second primary malignancies in patients with tonsillar squamous cell carcinoma.

Characteristics	Univariate analysis			Multivariate analysis		
	SHR	*95% CI*	P-Value	SHR	*95% CI*	P-Value
Ethnicity						
White people	Reference			Reference		
Black people	1.807	1.541-2.120	< 0.001	1.513	1.315-1.741	< 0.001
Others	0.967	0.721-1.310	0.830	0.854	0.654-1.113	0.240
Gender						
Male	Reference					
Female	1.080	0.938-1.230	0.290			
Radiation						
Yes	Reference			Reference		
None	1.300	1.130-1.490	< 0.001	1.697	1.490-1.933	< 0.001
Chemotherapy						
Yes	Reference					
None	0.925	0.826-1.040	0.180			
Surgery						
Yes	Reference			Reference		
None/Unknown	1.720	1.540-1.920	< 0.001	1.783	1.614-1.969	< 0.001
Marital status						
Married	Reference			Reference		
Single/Divorced	1.420	1.276-1.590	< 0.001	1.353	1.232-1.485	< 0.001
Unknown	1.220	0.959-1.550	0.110	1.226	1.010-1.489	0.040
Age (years)						
18-44	Reference			Reference		
45-54	1.730	1.230-2.430	< 0.001	1.399	1.078-1.815	0.012
55-64	2.530	1.820-3.540	< 0.001	1.912	1.481-2.468	< 0.001
> 64	3.440	2.460-4.800	< 0.001	2.776	2.145-3.594	< 0.001
Summary stage						
Localized	Reference			Reference		
Regional	0.907	0.852-0.963	< 0.001	0.831	0.720-0.959	< 0.001
Distant	1.755	1.457-2.120	< 0.001	1.432	1.219-1.683	< 0.001
Unknown	1.006	0.662-1.530	0.582	0.743	0.520-1.062	0.10
Primary site						
Tonsillar fossa	Reference			Reference		
Tonsillar pillar	0.896	0.705-1.137	0.370	0.905	0.747-1.096	0.310
Overlapping lesion of tonsil	0.779	0.448-1.356	0.380	0.760	0.502-1.151	0.200
Tonsil	0.802	0.694-0.927	0.002	0.811	0.720-0.914	< 0.001
Laterality						
Left	Reference					
Right	1.006	0.899-1.110	0.990			
Bilateral	1.330	0.696-2.560	0.390			
Others	1.170	0.532-2.560	0.700			

### 3.3 Nomogram construction and validation

**Figure 3:**
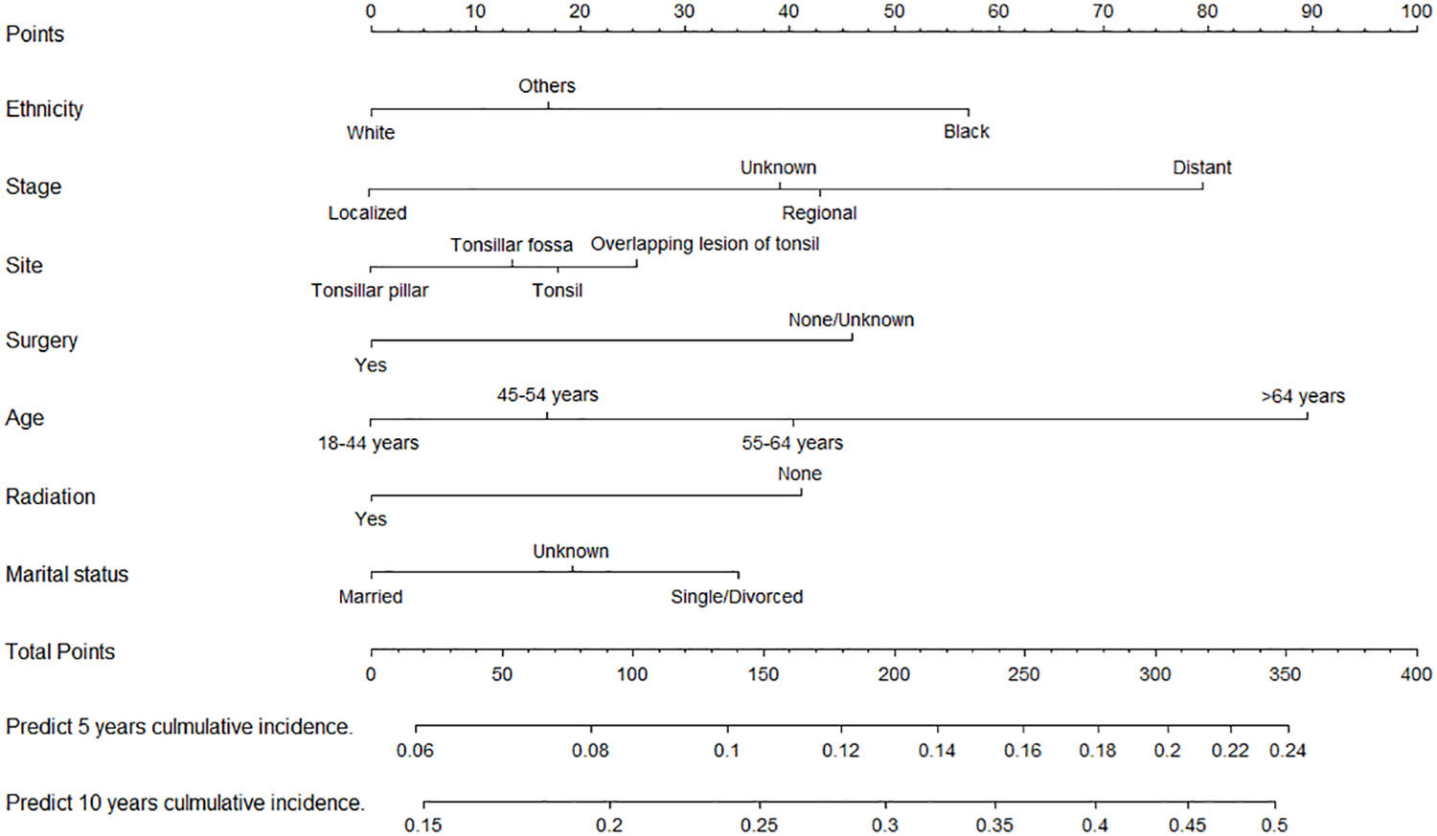
Nomogram for predicting the risk of 5- and 10-year tonsillar squamous cell carcinoma specific death.

**Figure 4:**
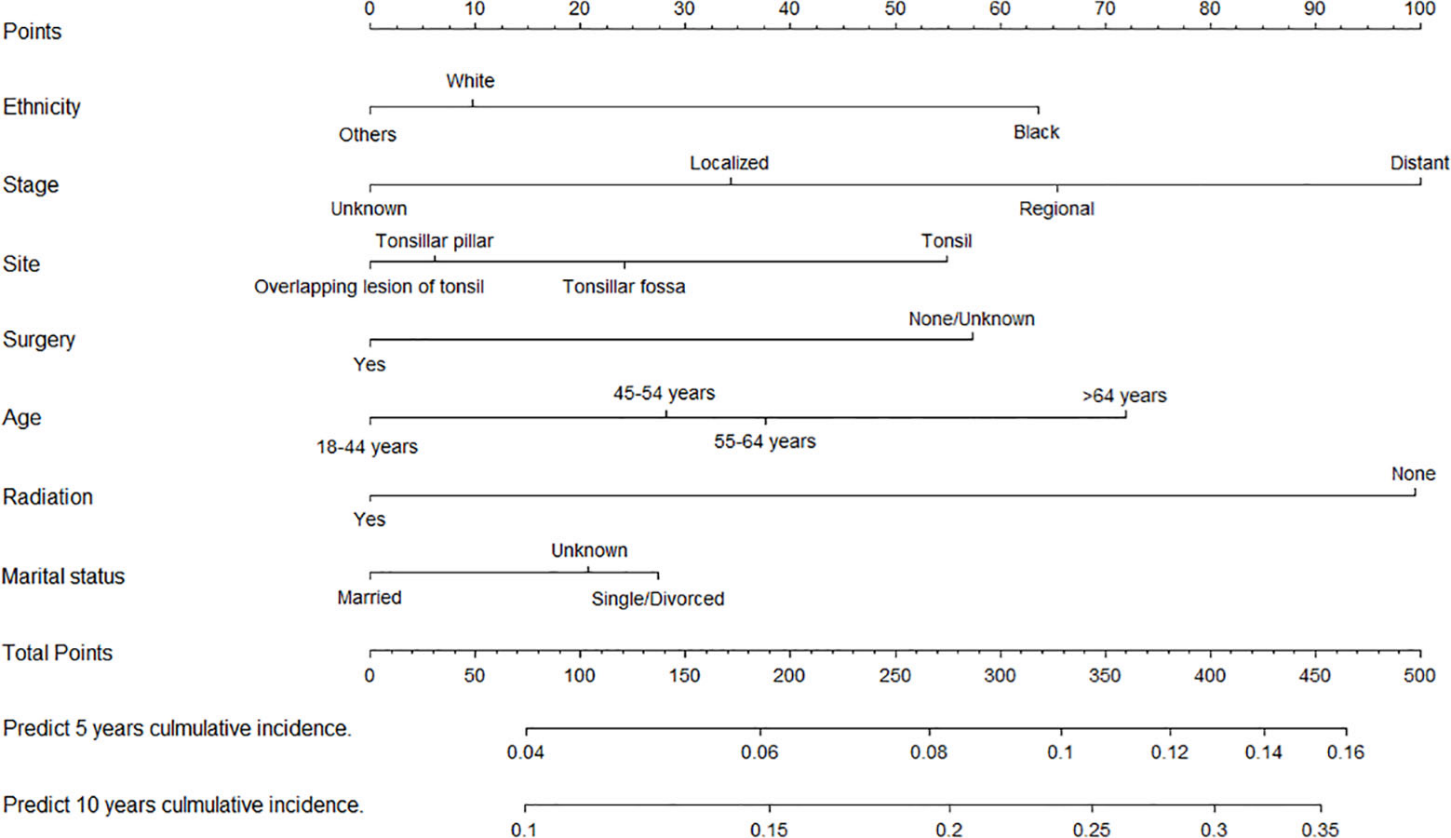
Nomogram for predicting 5- and 10-year risks of mortality from second primary malignancies in patients with tonsillar squamous cell carcinoma.

We constructed two nomograms for predicting 5- and 10-year TSD and SPM-related mortality by using the seven factors with statistical significance (*P* < 0.05) in multivariate analysis (**Figures 3**, **4**). Each factor is included in the nomogram as a line segment, and a numerical scale on the line segment indicates how much the factor affects risk. The scores of all factors for each patient were summed, and the total score corresponded to the probability of mortality from TSCC and SPM-related death at 5 and 10 years.

**Figure 5:**
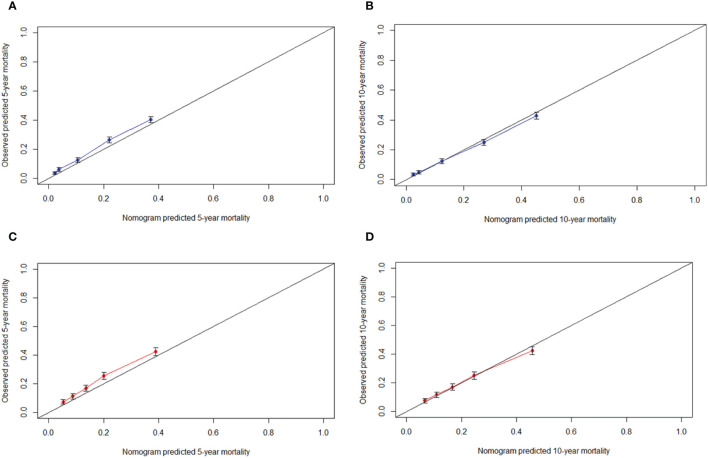
Calibration curve. **(A)** 5-year, **(B)** 10-year probabilities of tonsillar squamous cell carcinoma specific death in the training cohort. **(C)** 5-year, **(D)** 10-year probabilities of tonsillar squamous cell carcinoma specific death in the validation cohort. X-axis: predicted event probabilities by the nomogram. Y-axis: observed cumulative incidence for tonsillar squamous cell carcinoma death.

**Figure 6:**
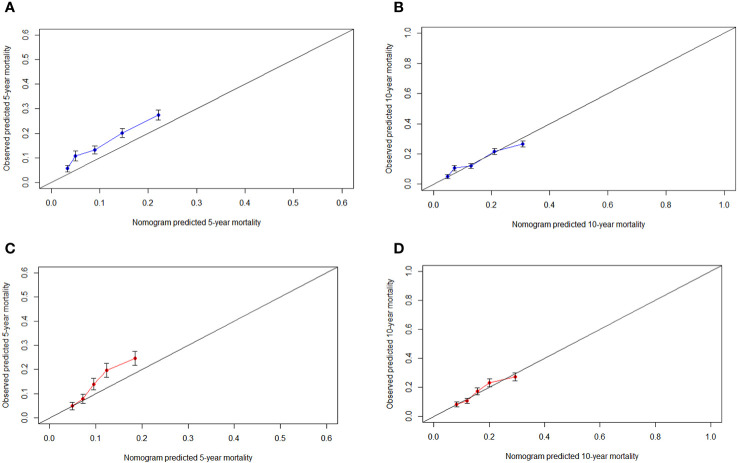
Calibration curve. **(A)** 5-year, **(B)** 10-year probabilities of second primary malignancies-related death in the training cohort. **(C)** 5-year, **(D)** 10-year probabilities of second primary malignancies-related death in the validation cohort. X-axis: predicted event probabilities by the nomogram. Y-axis: observed cumulative incidence for second primary malignancies death.

The *C*-indexes of the nomogram for predicting the 5- and 10-year probability of TSD were 0.813 and 0.812 in the training cohort and 0.794 and 0.794 in the validation cohort, respectively. Moreover, the *C*-indexes of the nomogram for predicting the 5- and 10-year probability of SPM-related mortality were 0.763 and 0.764 in the training cohort and 0.732 and 0.733 in the validation cohort, respectively. As shown in **Figures 5**, **6**, the calibration plots show good agreement between the observed probabilities and the nomogram probability predictions in the training and validation cohorts.

## 4 Discussion

To our knowledge, this study was the first to analyze the mortality patterns in patients with TSCC and establish the nomograms of TSD and SPM-related mortality under the framework of competing risk model. A previous study showed that the main sites of SPMs in head and neck carcinoid patients were the head and neck, larynx, and lungs (23). Another study showed that 10–40% of patients with head and neck squamous cell carcinoma developed SPMs, mainly located in the head and neck, esophagus, or lungs (24). Our study obtained a similar result: the most common sites of SPM that led to death in patients with TSCC were the lungs and larynx, followed by the esophagus and non-melanoma skin. Therefore, the SPMs in these sites should be carefully monitored. Moreover, elderly TSCC survivors who did not receive surgery or radiotherapy and TSCC patients who developed distant metastases might be at an increased risk of TSD and SPM-related death.

This study is also the first to formulate a competing risk nomogram for TSD in patients with TSCC. Only variables with clinical importance, high repeatability, and low time-varying effects were collected from the SEER database to balance comprehensiveness and comprehensibility of the prediction model (25). First of all, tumor stage has a significant impact on TSD risk, and the presence of distant metastasis stage has the greatest impact. According to the nomograms, patients with the same tumor stage can be assigned different point and have different survival outcome, indicating the rationality of our nomogram for prognostic prediction compared with the tumor stage (26). Moreover, therapeutic approaches, including radiotherapy and surgery, are important factors that affect TSD. Currently, radiotherapy is the preferred treatment modality for TSCC (27), whereas the role of chemotherapy has not been validated. Considering the continuous improvement of surgical methods (11), the survival outcome of TSCC is continuously improved, and the risk of TSD in patients without surgery increases (1). Age has always been a risk factor that affects tumor development and aggression (28). With increasing age, the immune function of elderly patients decreases, and the functions of tumor recognition and natural killer cancer cells continue to decline, thus accelerating cancer progression and resulting in a mortality rate that is higher than that of young people (29). In addition, black people had a higher risk of TSD than white people or other racial groups, possibly because black people have higher socioeconomic barriers to receiving timely and high-quality care than other ethnics (30). Finally, unmarried or divorced patients had a higher risk of TSD than married patients. Married patients may have better access to care than unmarried patients, and marital status may influence the stage of diagnosis in cancer patients, because spouses may encourage them to seek medical care for worrisome symptoms (31).

SPMs may lead to a reduced life expectancy for people with head and neck cancer (32). Considering the prolonged survival of TSCC patients, recurrence, metastasis, and SPMs are expected to increase. Various environmental factors, intrinsic genetic factors, and immune susceptibility may be important factors in the development of SPMs (33). In this study, we developed a competing risk nomogram for predicting SPMs-related mortality in TSCC patients, and several new discoveries were obtained. First, patients who have not undergone surgery or chemoradiotherapy have a much higher risk of SPM-related death than those who have undergone these treatments. Squamous cell carcinoma of the head and neck is sensitive to radiotherapy (34), which can also reduce the risk of SPMs (35). Radiotherapy or surgery is independently associated with better prognosis among patients with SPMs (36, 37). Second, elderly patients have a higher risk of SPM-related mortality than young patients, which may be related to treatment-related risk factors for SPMs (38). For example, elderly patients prone to suffer higher rates of postoperative complications, side effects, and longer stay in ICU than young patients (39), which may also put elderly patients at a higher risk of death than young patients. Elderly patients have a higher risk of SPMs than young patients possibly because of their lower ability to repair somatic DNA damage, thus accumulating potential mutations that promote carcinogenesis (40). Third, the later the tumor stage, the higher the risk of died from SPMs, indicating that patients with advanced TSCC are likely to develop SPMs (41). Bertolini et al. found that the majority (53.5%) of SPMs occurred in patients with stage IV head and neck cancers (23). Finally, similar to TSD, black people, and divorced or single patients have a higher risk of SPM-related mortality than other ethnics and married patients.

Considering the prolonged survival of TSCC patients, recurrence, metastasis, and SPMs are expected to increase. Various environmental factors, intrinsic genetic factors, and immune susceptibility may be important factors in the development of SPMs (33). Moreover, the phenomenon of ‘field cancerization’ may cause the oral cavity to be in direct contact with tobacco and alcohol carcinogens than other parts, thus increasing the risk of oral cancer patients suffering from various cancers, such as local recurrence and SPMs (42). Furthermore, HPV-related cancers may have a genetic predisposition to HPV infection, HPV transformation, and progression to HPV-related cancers (43, 44). This genetic predisposition also increases the risk of SPMs (45). Therefore, the monitoring of SPMs should be strengthened in TSCC patients, especially in the lung and bronchus, larynx, esophagus, and skin that are prone to develop SPMs (46).

The present study had revealed the mortality patterns of TSCC patients and developed competing risk nomograms with preferable discrimination and calibration, but some limitations should be acknowledged. First, this study is a retrospective study, and selection bias is unavoidable (47). Second, alcohol consumption and smoking habit might increase the risk of developing SPMs. However, due to insufficient information in the SEER database, we were unable to explore the effects of these factors on the mortality patterns of TSCC patients. Finally, the development and aggressiveness of TSCC is significantly associated with HPV infection, but the SEER database only recorded this information after 2010, leading to HPV infection status (positive or negative) was reported in only 30% of TSCC patients in this study and the rest (70%) were unknown. Considering that TSCC was a low-incidence cancer in the SEER database and this study was aimed to formulate prediction models, we have to include as many patients as possible to ensure the reliability and accuracy of the prediction model. Therefore, we decided not to include HPV infection in this study due to the high deletion rate.

## 5 Conclusion

In this population-based analysis, two competing risk nomograms were developed for predicting TSD and SPM-related mortality in TSCC patients. These nomograms have perfect performance in predictive accuracy and discriminative capability, which can be a useful tool to predict mortality risks from different causes at different time point in TSCC patients. Moreover, TSD and SPM-related death are the most common mortality patterns in TSCC patients. The lung and bronchus are the most common sites that lead to SPM-related death in TSCC patients, followed by the larynx and esophagus. These results suggest that the routine follow-up care of TSCC survivors should be extended to surveillance for SPMs to improve the clinical management and prognosis of TSCC patients. Considering the limitation of this study, further studies are needed to understand the underlying mechanisms of SPMs and to develop surveillance strategies for SPMs in patients with TSCC.

## Data availability statement

The datasets presented in this study can be found in online repositories. The names of the repository/repositories and accession number(s) can be found in the article/**Supplementary Material**.

## Ethics statement

The studies involving humans were approved by Surveillance, Epidemiology, and End Results Program. The studies were conducted in accordance with the local legislation and institutional requirements. The participants provided their written informed consent to participate in this study.

## Author contributions

Literature search: SY, XL, and JW. Study design: JW, DN, and JYW. Data collection: YZ, DN, and JW. Data analysis: JH, EY, and XH. Model construction: JW, YZ, and JYW. Manuscript writing and revising: JW, XL, DN, JH, EY, and YZ. All authors read and approved the final manuscript.
