# Supplementary material for: Mortality patterns of patients with tonsillar squamous cell carcinoma: a population-based study
Source: Front Endocrinol (Lausanne). 2023 Dec 7;14:1158593. doi: 10.3389/fendo.2023.1158593 (PMC10733501; doi:10.3389/fendo.2023.1158593)

**Supplementary Table 1.** Subgroup analyses by gender and ethnicity based on the competing risk model

| **Cause** | **Characteristics** | **Cumulative incidence (%)** | | **Gray’s test P-*value*** |
| --- | --- | --- | --- | --- |
|  |  | **5-year** | **10-year** |  |
| **TSCC** | ***Ethnicity*** |  |  | < 0.001 |
|  | White | 20.55 | 25.09 |  |
|  | Black | 39.65 | 44.98 |  |
|  | Others | 20.18 | 24.71 |  |
|  | ***Gender*** |  |  | < 0.001 |
|  | Male | 25.04 | 29.53 |  |
|  | Female | 21.28 | 25.91 |  |
| **SPMs** | ***Ethnicity*** |  |  | < 0.001 |
|  | White | 11.18 | 16.97 |  |
|  | Black | 26.93 | 35.28 |  |
|  | Others | 8.55 | 13.48 |  |
|  | ***Gender*** |  |  | < 0.001 |
|  | Male | 13.00 | 19.57 |  |
|  | Female | 11.99 | 17.80 |  |

TSCC, tonsillar squamous cell carcinoma; SPMs, second primary malignancies.

**Supplementary Figure 1.** Cumulative incidence estimates of deaths from tonsillar squamous cell carcinoma. (A) Ethnicity. (B) Gender. (C) Radiation. (D) Stage. (E) Surgery. (F) Marital status.


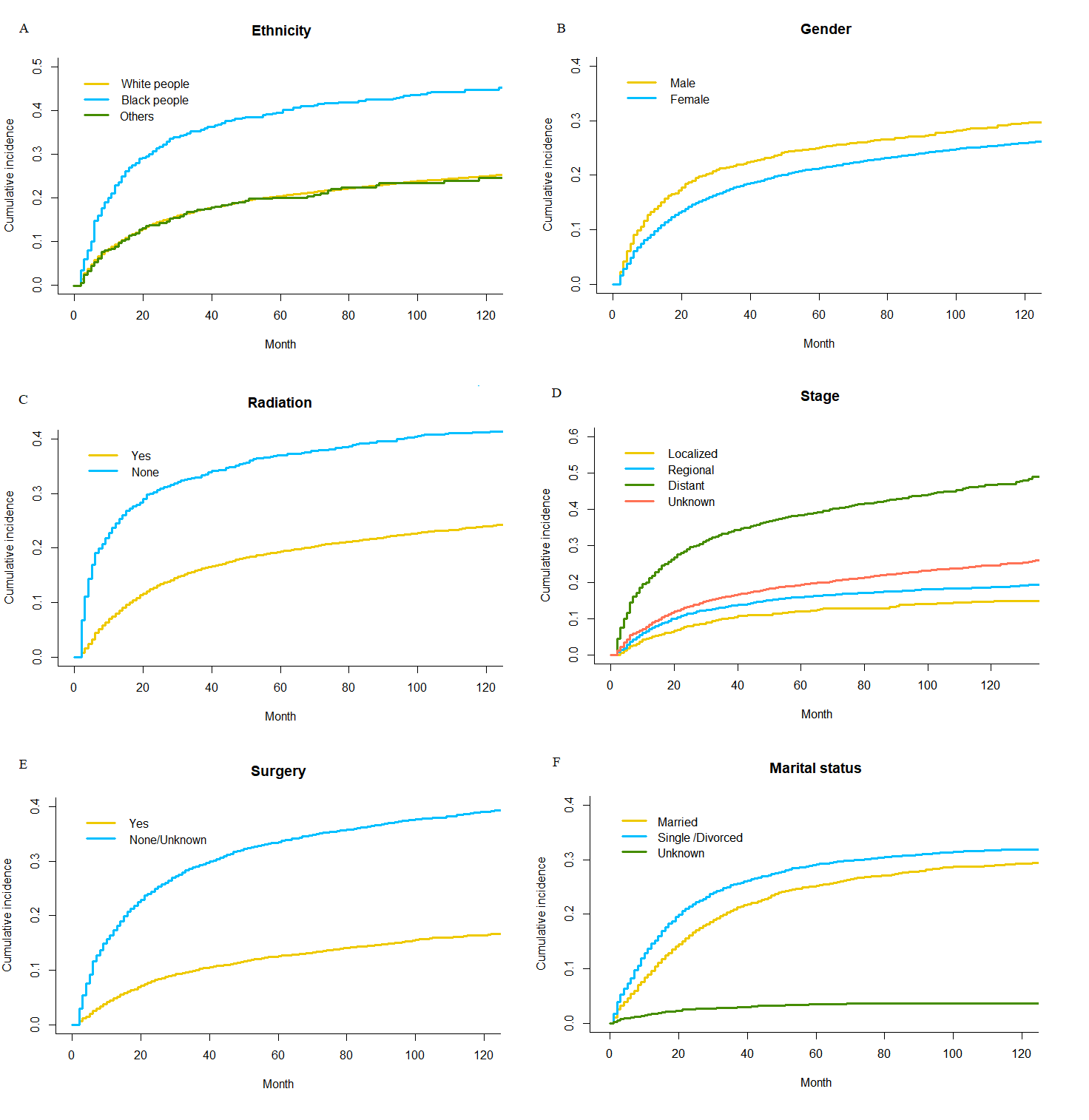


**Supplementary Figure 2.** Cumulative incidence estimates of deaths from second primary malignancies. (A) Ethnicity. (B) Gender. (C) Radiation. (D) Stage. (E) Surgery. (F) Marital status.


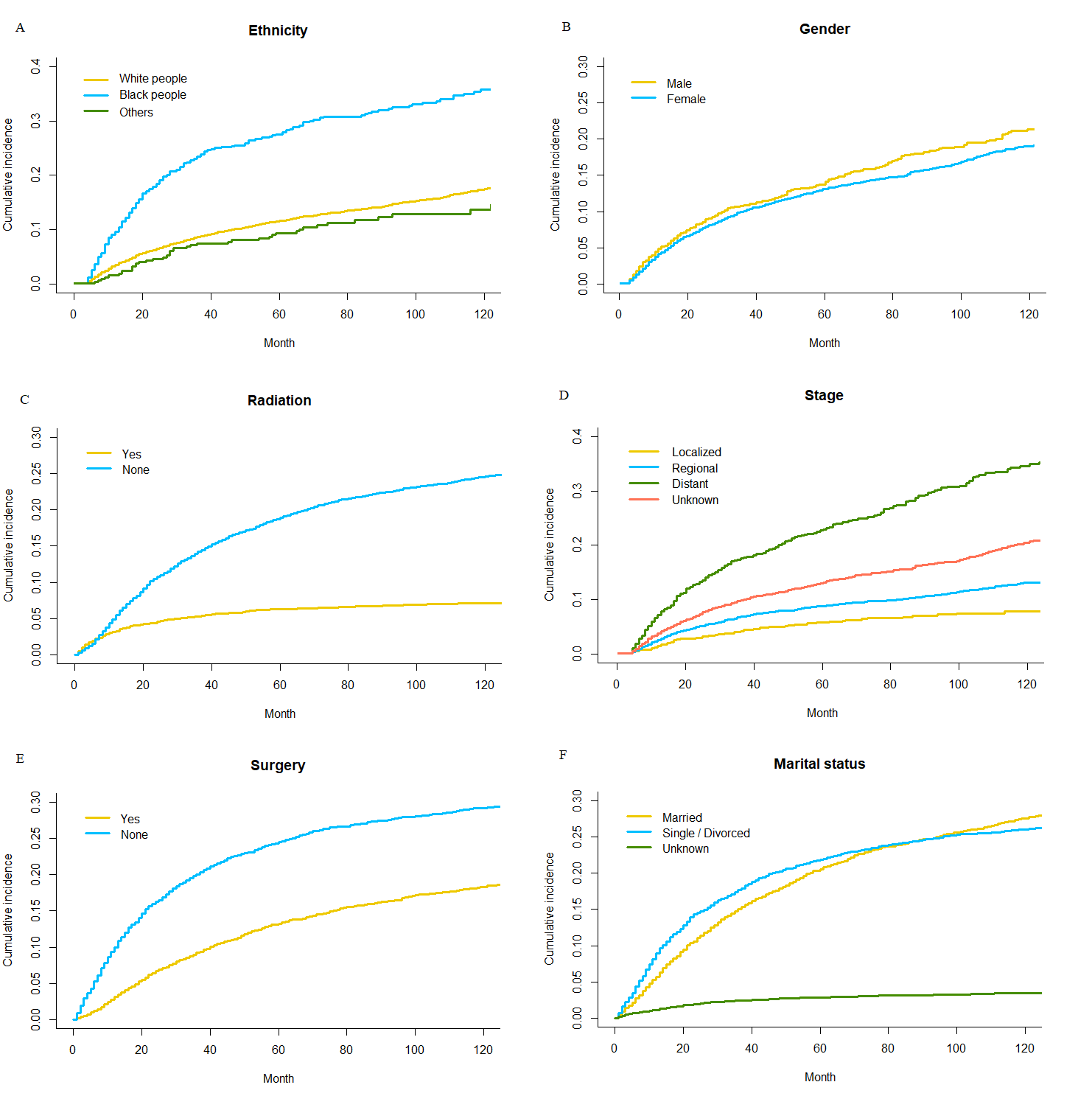

Supplement: Supplementary file 1 [file DataSheet_1.docx]
